# Supplementary material for: Suicidality among Chinese college students: A cross-sectional study across seven provinces
Source: PLoS One. 2020 Aug 21;15(8):e0237329. doi: 10.1371/journal.pone.0237329 (PMC7444487; doi:10.1371/journal.pone.0237329)
Supplement: Attachment — Submitted filename: PLoSOne_13072020_Suicidality of Students_Response.docx [file pone.0237329.s003.docx]

**Journal: PLOS ONE (PONE-D-20-00103)**

**Suicidality among Chinese college students: A cross-sectional study across seven provinces**

Dear Editor,

On behalf of my co-authors, I would like to convey our thanks for a further opportunity to revise our manuscript. Please find below our point-to-point response to the reviewer’s comments.

Thank you.

Warm regards

Dr. Siau Ching Sin

Reviewer’s comments:

Minor revisions required

The revised version of the paper is well-written and concise. Previous comments were reviewed by the authors and taken account of. It was identified that the response to reviewers were implemented in the latest copy of the study.

Very minor errors were identified and are as stated below.

The following are the line numbers and the recommendations.

Query #1. 79-95 I recommend ‘the collective body of evidence’ to be reformatted. It seems like a run-on sentence that takes a paragraph space on the paper.

Response: We have revised the paragraph as follows (ll. 79-89):

There is a collective body of evidence on the identification of risk and protective factors for suicide, which includes factors such as life stress and coping style [8], personality factors of impulsivity and aggression [9], and depression [9-12]. In addition, adverse developmental influences or events such as childhood adversity, divorce of parents, loss of a parent, and sexual abuse are also risk factors for increased suicidality [9-12]. Among Chinese college students, factors that have been found to influence suicidality are academic performance, academic stress, occupational future, recent conflicts with classmates, satisfaction with major, and the rupture of romantic relationships [12,13]. Finally, family influences such as parents’ educational level, family income, a history of suicide in the family, and originating from a rural background are also factors that are associated with suicidality among college students [14-19].

Query #2. 119-120 Recommend correcting the wording to ‘approximately a half an hour’.

Response: We have made the correction.

Query #3. 220 Recommend writing years old (y.o.) before using the abbreviation.

Response: We have made the correction.
